# Role of Cardiac Imaging in the Evaluation of Rheumatoid Arthiritis: A Systematic Analysis

**DOI:** 10.7759/cureus.113747

**Published:** 2026-07-31

**Authors:** Sourav S Panicker, Anirudh Danduboyina, Saatvika Reddy Borra, Sheethal S Panicker, Binay K Panjiyar

**Affiliations:** 1 Department of Cardiology, Dr. D. Y. Patil Medical College, Hospital and Research Centre, Pune, IND; 2 Department of Cardiology, Gandhi Medical College, Hyderabad, IND; 3 Department of Cardiology, Jawaharlal Nehru Medical College, Belagavi, IND; 4 Department of Cardiology, Sri Ramachandra Institute of Higher Education and Research, Chennai, IND; 5 Department of Internal Medicine, California Institute of Behavioral Neurosciences and Psychology, Fairfield, USA

**Keywords:** cardiac assessment, cardiac imaging, cardiac manifestations, echocardiography, rheumatoid arthritis

## Abstract

Rheumatoid arthritis (RA) is a chronic inflammatory autoimmune disease affecting the joints with extensive systemic inflammation. Cardiovascular deaths are one of the most common causes of death among RA patients. We performed an intensive literature review of abstracts, including the use of cardiac imaging in the evaluation of patients with rheumatoid arthritis from databases, including PubMed and Google Scholar libraries. Clinical trials, meta-analyses, randomized controlled trials, and systematic reviews were included. Review papers, animal studies, and studies without the role of cardiac imaging were excluded. We used quality appraisal tools including the Preferred Reporting Items for Systematic Reviews and Meta-Analyses (PRISMA) checklist, Cochrane Bias Tool, Newcastle-Ottawa Tool Scale, Critical Appraisal Skills Program (CASP) checklist, and the Scale for the Assessment of Narrative Review Articles (SANRA). Echocardiography, carotid ultrasound, myocardial stress single-photon emission-computed tomography (SPECT), and cardiac magnetic resonance imaging (MRI) were found to have good efficacy to detect ventricular dysfunction, subclinical atherosclerosis, perfusion defects, and myocardial fibrosis, respectively. Latest technological advancements in deep learning and machine learning-based algorithms could facilitate the incorporation of cardiac imaging for risk assessment in RA patients.

## Introduction and background

Rheumatoid arthritis (RA) is a chronic inflammatory autoimmune disease that primarily affects joints, with additional extra-articular manifestations [1]. The global prevalence of RA is between 0.24% and 1%. It appears to be more common in the developed nations [1]. RA can affect individuals of any age; however, incidence is higher after 40 years of age [2]. Women have a two to three times increased risk of developing RA than men [2]. Standardized mortality ratios for all-cause mortality as high as 1.5 to 1.6 have been recorded in RA patients, with the most common cause being cardiovascular diseases (CVD) [1,3].

The excessive systemic inflammation owing to RA plays a pivotal role in elevated cardiovascular events [4]. Inflammation promotes the pathogenesis and commencement of atherosclerosis, thus CVD in an individual [4]. Hence, there is an associated increase in the levels of proinflammatory molecules, including C-reactive protein (CRP), fibrinogen, and cytokines, in RA patients, contributing to structural abnormalities and endothelial dysfunction, in addition to changes in lipid levels, oxidative stress, and insulin resistance [4].

Pericarditis forms the most frequent cardiovascular manifestation of RA, while other cardiac pathologies comprise myocardial infarction, valvular heart disease, atherosclerosis, and arrhythmias [5]. Chronic inflammation plays a major role in the increased likelihood of heart failure (HF) and associated loss of life in patients diagnosed with RA [6]. Disabled left ventricular (LV) diastolic function with preserved LV ejection fraction (EF) and limited signs and symptoms of HF, unlike non-RA patients, makes meticulous evaluation of RA patients indispensable [7]. Drugs used in the management of RA are heterogeneous in their mode of action, dosage, and side effects [8]. Certain medications showcase detrimental cardiovascular side effects, perhaps by hampering the vascular repair mechanisms [8].

The present-day CV risk assessment models seem to be dysfunctional for RA patients [9]. The SCORE (Systematic COronary Risk Evaluation) calculator established for the general population does not incorporate non-traditional CVD risk factors and underestimates the future risk of CVD in RA patients by about 1.5 times [10]. Another risk prediction model, QResearch Cardiovascular Risk Algorithm (Qrisk) 2, evaluates the combined risk of fatal and non-fatal CV events. It includes RA as a risk factor with a multiplication factor of 1.4; however, it tends to overestimate the CVD risk in these patients [10].

The drawbacks of the aforementioned scores, along with the evolution in imaging techniques, caught the attention of researchers in the application of imaging modalities for cardiovascular risk assessment in RA patients [11]. Ultrasound of carotid arteries is a fundamental, safe, and easily accessible diagnostic imaging tool for atherosclerotic plaques that develop early in RA patients [9]. Cardiac magnetic resonance (CMR) can be used in RA patients with supposedly normal hearts to illustrate the underlying diffuse and focal myocardial fibrosis and inflammation [11]. CMR T1 mapping, in addition, is a non-invasive tissue characterization technique of added value, as they serve as a potential imaging biomarker for disease surveillance and the study of therapies aimed at reducing myocardial fibrosis in RA [11]. Certain cardiac pathologies like diastolic dysfunction, which may function as a precursor to systolic and diastolic HF in RA patients, can only be diagnosed by the application of imaging techniques like transthoracic echocardiography (TTE) [12]. Intra- or inter-observer variability noticed in the manual assessment of the image-based characterization for carotid images makes the artificial intelligence-based strategies and their automation significant in providing confidence to therapeutic policymakers and specialty physicians [13].

Through our review, we aim to provide an in-depth analysis of the existing literature regarding the role of cardiac imaging in the evaluation of patients with rheumatoid arthritis for screening, early diagnosis, personalized treatment regimens, and follow-up of patients to monitor the progression of cardiovascular morbidity due to RA.

## Review

Methods

**Figure 1:**
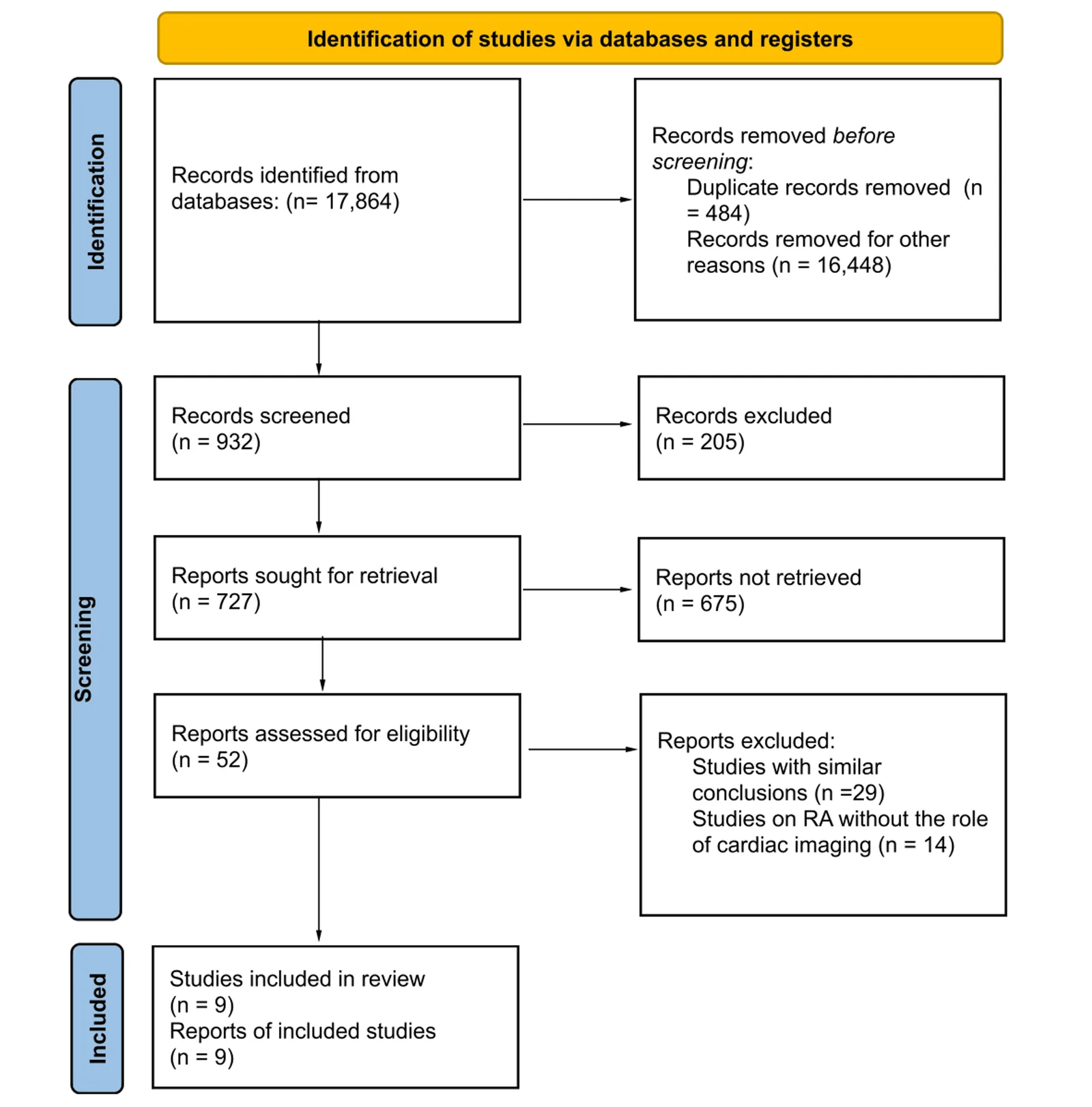
PRISMA flow diagram illustrating search strategy and study selection PRISMA: Preferred Reporting Items for Systematic Reviews and Meta-Analyses

This systematic review consolidates the literature on the role of cardiac imaging in the evaluation of RA. The review follows the guidelines for Preferred Reporting Items for Systematic Reviews and Meta-Analyses (PRISMA) 2020 [14] and strictly uses data obtained from published papers, thus precluding the need for ethical approval (Figure 1).

Systematic Literature Search and Study Selection

We performed a detailed search for related studies using PubMed, including Medline and Google Scholar as our databases. We narrowed down the studies mentioned under clinical trials, meta-analyses, randomized controlled trials, and systematic reviews on PubMed. In addition, we continued searching for other studies that satisfied our inclusion criteria on Google Scholar.

We individually analyzed a set of abstracts for inclusion using specific criteria. The criteria included using cardiac imaging in the evaluation of patients with rheumatoid arthritis and a well-described clinical cohort in the study. We excluded animal studies and studies involving rheumatoid arthritis without the role of cardiac imaging. A dual review was conducted independently by four reviewers, and controversies were sorted through discussions.

Inclusion and Exclusion Criteria

We established the definite criteria for the inclusion and exclusion of participants to attain unbiased study results. Our criteria are summarized in Table 1.

**Table 1:** Criteria for literature search

	Inclusion Criteria	Exclusion Criteria
1.	Human studies	Animal studies
2.	Studies from June 2013 to June 2023	
3.	English articles	Non-English articles
4.	Gender: Males and females	
5.	Age: Above 19 years of age	Age: 19 years of age or below
6.	Free articles	Articles to be purchased
7.		Studies on rheumatoid arthritis without the role of cardiac imaging

Search Strategy

The population, intervention/condition, control/comparison, and outcome (PICO) criteria were employed to perform an intensive literature review on databases including PubMed (including Medline) and Google Scholar Libraries, using specific keywords, which consisted of cardiac assessment, cardiac manifestations, echocardiography, cardiac imaging, and rheumatoid arthritis. The medical subject heading (MeSH) approach was utilized in PubMed (including Medline) and Google Scholar to create an extensive search strategy (Table 2).

**Table 2:** Search strategy

	Database	Search strategy	Search results
1.	PubMed (including Medline)	(((Rheumatoid arthritis[Title/Abstract]) OR (cardiac imaging[MeSH Terms])) OR (evaluation[MeSH Terms])) AND (("2013/06/01"[Date - Publication] : "2023/06/30"[Date - Publication]))	3933
2.	Google Scholar	Rheumatoid arthritis AND cardiac imaging OR evaluation	1,36,000

Quality Appraisal

To establish the credibility of our chosen papers, we employed various quality assessment tools. We followed the PRISMA checklist and Cochrane bias tool assessment for systematic reviews and randomized clinical trials, respectively. Non-randomized clinical trials were assessed using the Newcastle-Ottawa Tool Scale. We analyzed the quality of qualitative studies using the Critical Appraisal Skills Program (CASP) checklist [15]. To avoid any confusion in the classification, we utilized the scale for the assessment of narrative review articles (SANRA) [16] to evaluate the article's quality (Table 3).

**Table 3:** Quality appraisal tools PRISMA: Preferred Reporting Items for Systematic Reviews and Meta-Analyses; SANRA: Scale for the assessment of non-systematic review articles; RCT: randomized controlled studies.

Quality Appraisal Tool	Types of studies used
Cochrane Bias Tool Assessment	Randomized Control Trials
Newcastle-Ottawa Tool	Non-RCT and Observational studies
PRISMA Checklist	Systematic Reviews
SANRA Checklist	Any other without a clear method section

Results

We extracted 17,864 articles after searching the aforementioned databases. We then meticulously reviewed each paper, after which 16,932 articles were removed in view of duplicates and not meeting our inclusion criteria. From the remaining 932 studies, we did not use 880 articles due to unsuitable titles and abstracts. We precisely analyzed the remaining 52 studies and further excluded 43 studies due to similar conclusions or deviating outcomes. Lastly, we conducted a profound quality analysis on the remaining nine papers, all of which fulfilled our criteria. These nine studies are summarized in our final systematic review (Table 4).

**Table 4:** Summary of studies RA: Rheumatoid arthritis; LV: left ventricle; RV: right ventricle; CVD: cardiovascular disease; CMR: cardiovascular magnetic resonance.

Author /Year	Country	Study Design	Database	Conclusion
Corrao et al. (2013) [17]	Italy	Systematic review and meta analysis	PubMed-MedLine, Cochrane Central Register of Controlled Trials (CENTRAL)	Highlights the prevalence of pericardial and valvular involvement in RA patients asymptomatic for cardiac disease.
Myasoedova et al. (2013) [6]	USA	Cross sectional study	Rochester Epidemiology Project (REP)	RA is associated with abnormal left ventricular remodeling, particularly concentric remodeling, even in patients without heart failure.
Aslam et al. (2013) [12]	USA	Systematic review and meta analysis	Medline, CENTRAL, Web of Science	RA patients had higher chances to demonstrate echocardiographic evidence of diastolic dysfunction, elevated systolic pulmonary artery pressure, and larger left atrial size.
Fine et al. (2013) [7]	USA	Retrospective cohort study	Rochester Epidemiology Project (REP)	Speckle tracking echocardiography revealed global longitudinal LV and RV strain irregularities in RA patients, which corresponded to RA intensity.
Joyce et al. (2013) [18]	Ireland Netherlands	Case based literature review	Mater Misericordiae University Hospital	Regular screening of RA patients taking hydroxychloroquine with transthoracic echocardiography and 12-lead electrocardiogram could timely reveal drug-induced cardiomyopathies and conduction abnormalities.
Corrales et al. (2013) [19]	Spain	Cross-sectional study	104 RA patients recruited from Hospital Universitario Marqués de Valdecilla (Santander, Northern Spain)	Carotid ultrasonography is more sensitive than coronary artery calcification score (CACS) for early diagnosis of subclinical atherosclerosis in RA patients.
Bernardes et al. (2017) [20]	Portugal	Cross-sectional study	PubMed, Google Scholar, Crossref	Myocardial perfusion single photon emission-computed tomography revealed subclinical myocardial perfusion irregularities in RA patients.
Ntusi et al. (2015) [11]	UK, South Africa, Greece	Prospective case-control study	Google Scholar, CrossRef, Scopus	CMR T1 mapping can be utilized to evaluate and monitor the progression of myocardial fibrosis and edema in RA patients.
Khanna et al. (2019) [13]	USA, UK, Greece, Italy, India	Systematic review	PubMed	The employment of B-mode ultrasound for carotid atherosclerosis and machine learning for risk stratification in patients with rheumatoid arthritis aids CVD risk stratification.

Discussion

Cardiac involvement of RA ranges from the most common manifestation of pericarditis to accelerated atherosclerosis, causing premature mortality in RA patients [17]. A systematic review and meta-analysis conducted by Corrao et al. [17] noted significant cardiac modifications identified using echocardiography in RA patients who lacked clinical signs and symptoms pointing towards cardiac involvement. They concluded that pericardial effusion, valvular thickening and nodules, isolated valvular insufficiency (especially mitral and aortic valves), aortic root alteration, and structural and functional left ventricular changes, including increased left ventricular mass and diastolic dysfunction causing congestive heart failure (CHF), are characteristic findings of RA patients asymptomatic for cardiac disease, making it a “silent rheumatoid heart disease” [17]. They noted 10 times higher risk of pericardial effusion and valvular nodules in RA patients compared to their controls [17].

A cross-sectional study conducted by Myasoedova et al. [6] on RA patients using two-dimensional/Doppler echocardiography revealed that the prevalence of abnormal left ventricular (LV) geometry is 1.4 times higher in RA patients, especially concentric remodeling, which amounted to 44% in RA patients compared to 19.2% among non-RA individuals, despite adjusting traditional risk factors for cardiovascular disease (CVD). LV remodeling is known to be a precursor of CHF, thus leading to increased morbidity and mortality among RA patients [6]. LV Mass Index (LVMI) seems to vary among RA patients. A study showed lower LVMI in RA patients using corticosteroids [6], while another study showed a higher LVMI among RA patients [12]. In contrast, another study showed no significant difference [7] between the LVMI of RA and non-RA groups. These contradictory results could be owing to the serial changes occurring in the myocardium due to the ongoing disease process of RA and the effects of medications [6].

Various studies [7,12,21] have documented an increased risk of diastolic dysfunction with preserved ejection fraction among RA patients. A systematic review and meta-analysis conducted by Aslam et al. [12] revealed LV diastolic dysfunction (LVDD) to be more likely among RA patients than their controls, as evidenced by the most common echocardiographic findings of increased isovolumetric relaxation time, higher mean left atrial (LA) diameter, and elevated pulmonary artery pressure (PAP). The bigger LA itself poses a higher risk for atrial fibrillation in RA patients [12]. The dyspnoea and fatigue in certain RA patients could be attributed to the elevated PAP and cardiac involvement in RA [12]. The authors also noted right ventricular (RV) dysfunction among RA patients in three out of four studies [12]. 

A study by Fine et al. [7] suggested strain imaging of the myocardium using speckle-tracking echocardiography (STE) detects subclinical CVD among RA patients with normal ejection fraction. The study revealed global LV and RV peak systolic strain impairment among RA patients compared to their normal controls despite adjusting for the traditional CVD risk factors. RA strain impairment had a characteristic imaging pattern of impaired apical strain values, predominantly affecting the anteroseptum and posterior wall of the LV and both the free wall and septum of the RV. Strain values in RA patients correlated with RA severity markers like the Health Assessment Questionnaire (HAQ) score and the need for treatment. Strain impairment did not have a correlation with diastolic dysfunction and was found even among patients with no diastolic dysfunction, making it more sensitive to mechanical LV dysfunction. The automation and lower imaging frame rate requirement of STE in comparison to Doppler-based echocardiography make it a favorable cardiac strain screening imaging modality to detect early CVD [7].

Drugs approved for rheumatoid arthritis are expected to improve cardiovascular outcomes in RA by reducing inflammation, as exhibited by methotrexate [22]. However, multiple studies have demonstrated that most other anti-rheumatic drugs exhibit negative cardiovascular outcomes depending on the dosage and duration of administration due to adverse effects on traditional risk factors [8,16,23]. Multiple case reports [16,23] have documented the incidence of rapidly evolving drug-induced non-ischemic cardiomyopathy and conduction abnormalities, including atrio-ventricular and bundle branch block among RA patients treated with hydroxychloroquine, a major drug used in the treatment of RA. Prognosis in these patients can range from partial or complete resolution of cardiac involvement to cardiac transplantation or death despite immediate discontinuation of the drug [16]. Transthoracic echocardiography (TTE) and 12-lead electrocardiogram (EKG) play a key role in early diagnosis. TTE shows a characteristic finding of diffusely thickened ventricular walls with other common findings, including concentric ventricular hypertrophy, restrictive diastolic dysfunction, biatrial enlargement, and systolic dysfunction with reduced LV ejection fraction at later stages [18]. Cardiac magnetic resonance (CMR) imaging also plays a pivotal role in being the reference standard for the evaluation of LV and RV morphology and function. It rules out differential diagnoses, including myocarditis, sarcoidosis, amyloidosis and also determines the degree of cardiac fibrosis using delayed gadolinium enhancement (DGE) [16]. The elevated risk of fatal cardiac involvement with the potential of irreversibility warrants the regular screening of these patients with a 12-lead EKG and TTE, especially for high-risk patients with pre-existing risk factors [16,21]. The prognostic role of CMR imaging to determine timely interventions, including devices and cardiac transplantation, could be studied with further research [16,23].

The European League Against Rheumatism (EULAR) recommendations underscore the increased cardiovascular risk among RA patients and the urgent need for targeted CVD risk management [10]. Studies [10,19,24] reveal the scarcity of RA-specific CVD risk prediction algorithms and confirm that the existing CVD risk prediction scores for the general population do not effectively predict cardiovascular risk among RA patients. RA patients demonstrate increased risk for subclinical atherosclerosis in comparison to the general population [25]. A study by Corrales et al. [19] affirmed the greater sensitivity of carotid ultrasonography measuring carotid artery intima media thickness (cIMT) and the presence of plaques over Coronary Artery Calcification Score (CACS) determined by multi-detector CT (MDCT) scan for early detection of subclinical atherosclerosis, especially in RA patients with intermediate risk for CV events according to the Systematic Coronary Risk Evaluation modified according to EULAR recommendations (mSCORE). The study revealed a correlation between the mSCORE and the CACS and also between the CACS and the cIMT. However, a number of patients with normal or low to moderate CACS (CACS<100) exhibited severe ultrasonography findings, especially carotid plaques, which are predictive of a CV event in these patients. In addition, only 20% patients with moderate mSCORE showed CACS>100 (high CV risk), while 85% of these patients demonstrated carotid plaques on ultrasonography. But 57.5% of patients with normal CACS (CACS=0) also showed the presence of coronary plaques on ultrasonography, which is suggestive of high cardiovascular risk. This suggests that carotid ultrasonography could be used as a noninvasive, cost-effective method to detect subclinical atherosclerosis and high CV risk in RA patients with moderate mSCORE and normal to moderate CACS [19]. The individual sensitivity of mSCORE, CACS, and carotid ultrasonography to detect high CV risk in RA patients is 19.4%, 23.6%, and 97.2%, respectively [19]. The EULAR task force recommends considering screening of RA patients for subclinical atherosclerotic plaques using carotid ultrasound and CVD risk assessment at least once in five years and following major changes in antirheumatic therapy [10]. This will aid physicians in adjusting the dose of nonsteroidal anti-inflammatory drugs and steroids in RA patients with high CV risk and also allow early initiation of lifestyle modifications and preventive treatment for the best CV outcomes in RA patients [10].

RA patients without CAD have a higher prevalence, extent, and severity of all types of coronary plaque [25]. A study [20] by Bernard et al. revealed the utility of myocardial stress single-photon emission-computed tomography (SPECT) in detecting subclinical perfusion defects. MPS showed myocardial perfusion defects (summed stress score (SSS) ≥4) in 25% of patients; mild defects (SSS ≥4 and ≤8) in 18.5% of patients, moderate (SSS≥4 and and ≤8) in 5.3% of patients, and severe (SSS>13) in 1% of the patients. LV ejection fraction (LVEF) was below 45% in 4.76% of the patients. Two of these patients died in less than five years from cardiovascular causes. The perfusion defects were unique (53%) or multiple (47%), with no peculiar distribution. There was no relationship between the myocardial perfusion defects and patient characteristics or traditional cardiovascular risk factors. In addition, it was noted that B-type natriuretic peptide (BNP) levels more than 100 pg/mL were associated with the presence of myocardial perfusion scan (MPS) defects. It appears prudent to include cardiac imaging studies like MPS in primary prevention strategies, which is a noninvasive test that assesses macro- and microvascular disease.

Myocardial dysfunction in RA is a result of various pathophysiological mechanisms, including myocardial fibrosis and inflammation [11]. Various studies have demonstrated the utility of cardiac MRI (CMR) in diagnosing clinical and subclinical myocardial fibrosis and myocarditis using T1 mapping and extracellular volume (ECV) assessment in patients with connective tissue diseases, including RA [26,27]. A study by Ntusi et al. [11] revealed late gadolinium enhancement (LGE), corresponding to areas of focal myocardial fibrosis, in 46% of RA patients with a non-ischemic pattern of scattered mid-wall enhancement, compared to none in their control group. In addition, RA patients (10%) exhibited focal areas of myocardial edema. They had higher native T1 values and ECV (973±27 ms vs 961±18 ms, p<0.03, and 30.3±3.4% vs 27.9±2%, p< 0.001, respectively), demonstrating subclinical fibrosis or inflammation. Peak systolic and diastolic circumferential strain were also noted to be impaired in RA patients. T1 mapping could thus become a valuable tool to assess cardiac status in RA patients.

Cardiovascular risk predictive models play a vital role in stratifying RA patients. Various studies [13] have shown that various risk predictive models, including the Framingham Risk Score (FRS) and the Systematic COronary Risk Evaluation (SCORE), which used traditional CV risk factors, underestimated CVD risk in RA patients. Image-based, intelligence-based risk algorithms provide an alternative to predict CVD risk in RA patients. Machine learning-based tissue characterization using texture- or morphology-based features from carotid ultrasound images to characterize the vessel wall revealed subclinical atherosclerosis. Plaque composition, echogenicity, and echolucency were noted to be strong predictors of CVD and stroke risk [13]. A study by Lekadir et al. [28] used deep learning-based features to characterize the carotid ultrasound images into classes including lipid, fibrous, and calcified plaques. With advancements in technology, deep learning-based tissue characterization of images is context-based and does not depend on previous feature information, aiding characterization of complex overlapping image features like in RA.

## Conclusions

RA is associated with a significant burden of clinical and subclinical cardiovascular disease, including left ventricular remodeling, valvular involvement, and pericardial disease. Echocardiography revealed the prevalence of bilateral ventricular strain patterns among asymptomatic RA patients and could diagnose drug-related cardiac side effects among patients receiving treatment for RA. Studies reveal that cardiac imaging, including carotid US, myocardial SPECT, and cardiac MRI, has good efficacy to detect subclinical atherosclerosis, perfusion defects, and myocardial fibrosis, respectively. They have the potential to become the standard of care for cardiac evaluation in RA patients, at least in the high-risk group. Latest technological advancements in deep learning and machine learning-based algorithms can facilitate the incorporation of cardiac imaging for risk assessment in RA patients.
